# Evaluation of a novel caries detecting oral rinse

**DOI:** 10.1038/s41405-023-00134-y

**Published:** 2023-03-20

**Authors:** Bennett Tochukwu Amaechi, Thais Santiago Phillips, Betty Isabel Perozo, Yuko Kataoka, Fatemeh Movaghari Pour, Rayane Farah, Amos Chinedu Obiefuna, Moshtagh Rashid Farokhi

**Affiliations:** 1 Department of Comprehensive Dentistry, School of Dentistry, University of Texas Health San Antonio, 7703 Floyd Curl Drive, San Antonio, TX 78229-3900 USA; 2 ANCOSTAT Statistical Consulting, San Antonio, TX 78245 USA

**Keywords:** Minimal intervention dentistry, Enamel, Caries risk assessment, Restorative dentistry

## Abstract

**Aim:**

LumiCare™ Caries Detection Rinse (LC Rinse), a starch-based rinse, illuminates active initial caries (positive response) using dental curing light, thus augmenting the dentist’s visual examination. This clinical study investigated if active caries as assessed by the International Caries Detection and Assessment System (ICDAS) were more likely to have positive LC Rinse response than sound surfaces and inactive caries.

**Methods:**

25 subjects participated in the study. Caries was assessed on selected teeth and the entire dentition, firstly using ICDAS and then by fluorescence evaluation after LC Rinse application. Data were statistically analyzed using Diagnostic Odds Ratio (OR) and Chi-square test *X*^2^ (α = 0.05). Sensitivity (Se), specificity (Sp), and Diagnostic accuracy (DA) were calculated.

**Results:**

With selected teeth, active caries were 638.6 times (60.05 with full dentition) more likely to have positive LC Rinse response than sound surfaces and inactive caries combined (X^2^, *p* < 0.01) and 191.67 times (18.35 with full dentition) than inactive lesions only (X^2^, *p* < 0.01). With combined sound surfaces and inactive caries, Se, Sp, and DA of LC Rinse assessment were 0.94, 0.98, and 0.96 respectively.

**Conclusions:**

LC Rinse can distinguish between active caries, inactive caries and hypomineralization, and can augment caries detection with high sensitivity, specificity, and diagnostic accuracy.

## Introduction

Current caries detection is normally performed by visual-tactile examination [1–3]. However, this is subjective in nature and has limited sensitivity, and tactile probing with a dental explorer can risk cavitation of otherwise remineralizable lesions [3–15]. Caries are also commonly detected radiographically, enabling clinicians to detect proximal lesions earlier than visual detection alone. However, there are concerns regarding the exposure to ionizing radiation and radiographs are not suitable for detecting early enamel caries on occlusal surfaces [6, 7, 11, 12, 16]. These limitations of the visual-tactile and radiographic caries detection and diagnosis have led to efforts to facilitate objective detection of dental caries, such as laser or light-induced fluorescence, transillumination, electrical impedance, bioluminescence, and others [17, 18]. While these methods, in conjunction with visual inspection and comprehensive caries risk analysis, can assist in improving treatment decisions, and ultimately, patient outcomes, caries diagnosis leading to effective treatment decisions remains a challenge for the profession [1, 19–22].

It is known that some initial caries lesions can become inactive or “arrested” when surface porosity is reduced by mineral deposition, and therefore may not require treatment. Presently, the distinction of arrested lesions from active lesions is based on ICDAS and Nyvad criteria [6, 10, 20–22], which are visual-tactile and subjective. The whitish appearance of initial caries also needs to be differentiated from developmental hypomineralization, a diagnosis that is made clinically based on observable features. Hence there is a need for a technology that can objectively detect initial caries and distinguish active carious white spot lesions from arrested caries lesions and developmental hypomineralization.

**Fig. 1:**
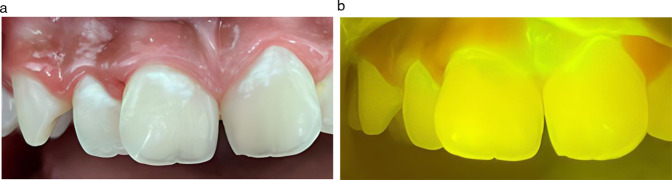
Photographic image of fluorescence illumination of active initial caries lesions. The same active initial caries lesions before (**a**) and after (**b**) rinse application.

GreenMark Biomedical Inc. has developed LumiCare™ Caries Detection Rinse (LC Rinse), a diagnostic oral rinse containing proprietary fluorescent starch nanoparticles that illuminates active initial caries lesions (positive LC Rinse response) using a dental curing lamp, thus augmenting the dentist’s visual examination (Fig. 1a, b) [23, 24]. In extracted teeth, LC Rinse demonstrated high reproducibility and accuracy for occlusal caries detection, with high sensitivity and specificity compared to histology [24]. A recent study has identified the utility of LC Rinse in combination with Artificial Intelligence (AI)/Computer Vision as an objective tool for caries diagnosis [25]. The current FDA-cleared indication for use of LC Rinse is to aid dental professionals in the visualization of caries lesions.

The objectives of the present clinical study were: (1) to determine if tooth surfaces with active caries lesions as assessed by ICDAS were more likely to have positive LC Rinse response compared to sound surfaces, inactive initial caries, or hypomineralization and (2) to determine the duration of LC Rinse test positivity in active caries lesions (i.e., to determine “assessment window”).

## Materials and methods

This clinical study was a cross-sectional (non-interventional), single-site, single-visit evaluation of an FDA cleared caries detection device conducted at our clinical research facility and approved by our Institutional Review Board (Approval #: 20210608HU). The study was conducted in accordance with ethical standards outlined in the 1964 Declaration of Helsinki and its later amendments, and in compliance with the International Conference on Harmonization Good Clinical Practice Guidelines. Written informed consent was obtained from all participants prior to their participation in the study.

### Participants recruitment

Prior to the study, a Cariologist and expert in caries detection and diagnosis (AFZ, acknowledged) served as a benchmark examiner, trained and calibrated the study clinical examiner (TSP) on ICDAS caries detection criteria and Nyvad criteria for distinguishing active caries from inactive caries and other differential diagnoses, such as hypomineralization. Inter- and intra-examiner reliability was assessed using weighted kappa scores. The clinical examiner’s intra-examiner and inter-examiner agreement values (weighted κ value) for training and calibration on ICDAS caries detection criteria were 0.95 and 0.62, respectively, which met the pre-set threshold (≥0.81 for intra-examiner and ≥0.61 for inter-examiner) for acceptability.

Twenty-five subjects aged 10 to 80 years with a minimum of 20 teeth with a range of active initial caries were included in this study. Participants were selected based on having a minimum of two teeth with active and two teeth with inactive initial caries (ICDAS 1 or 2) on unrestored surfaces and two sound surfaces. These lesion categories were selected based on ICDAS and Nyvad caries detection and diagnosis criteria [6, 10, 20–22]. Active lesions were clinically assessed as those in which the enamel surface was whitish/yellowish opaque (chalky) with loss of luster when air-dried, and which felt rough when the tip of a dental explorer was moved gently across the surface [20–22]. In contrast, the enamel surface of inactive lesions is more likely to appear shiny with whitish, brownish, or black color and feel rough or sometimes smooth [20–22]. Only occlusal and smooth surfaces were assessed. Teeth with hypoplasia, fractures, dentinogenesis imperfecta, and other conditions affecting the structural health of the enamel were excluded. Subject exclusion criteria included tetracycline stain, amelogenesis imperfecta, oral or systemic conditions preventing care in an ambulatory setting, receiving remineralization treatment, inability to use an oral rinse, tooth bleaching within the past six months, fixed orthodontic appliances, pregnant or nursing.

### Study procedures

Initial telephone or in-person screening preceded an enrolment visit in which eligibility was confirmed according to the inclusion and exclusion criteria, and informed consent was obtained. The patient underwent professional dental cleaning after enrolment to remove dental plaque and calculus. Following cleaning, a full mouth clinical examination consisting of hard and soft tissue examinations was performed to detect caries lesions and any existing soft tissue lesions. Caries detection was conducted using a reference method (ICDAS-II assessment) and the index test (LC Rinse) as described below.

#### Reference method: visual caries examination (ICDAS-II assessment) and selection of test and control sites

A clinical full dentition caries evaluation was performed by the study examiner to detect caries and stage lesion severity by ICDAS criteria (Table 1) [6]. The lesion activity assessment criteria of Nyvad et al. were used to distinguish between active and inactive lesions [20–22]. Teeth with hypomineralization, when present, were also recorded. The diagnosis of developmental hypomineralization was established if the opacity appears lying underneath a translucent enamel layer with glossy surface when air-dried, feels smooth when the tip of the probe is moved gently across the surface, had a color varying from white to yellow, and located mainly in self-cleansing areas (Table 2) [26, 27].

**Table 1:** International Caries Detection and Assessment System (ICDAS) scoring criteria [6].

ICDAS codes	Caries severity
0	Sound tooth surface
1	First visual change (opacity or discoloration) in enamel not or minimally visible on the wet surface but distinctly visible after air drying
2	Distinct visual change (opacity or discoloration) in enamel, visible without air drying
3	Localized enamel breakdown, no dentin visible
4	Dentin shadow (not cavitated into dentin)
5	Distinct cavity with visible dentin
6	Extensive distinct cavity with visible dentin

**Table 2:** Differentiating the causes of white spot lesions as caries versus developmental hypomineralization [26, 27].

Criteria for Distinction	Early caries	Developmental hypomineralization
Appearance	Opaque, chalky and dull (loss of lustre) surface when air-dried	Glossy surface when air-dried
Texture	Feels rough when the tip of the explorer is moved gently across surface	Feels smooth when the tip of the explorer is moved gently across the surface
Shape	Elliptical or crescent shaped	Lines resembling pencil shading
Area affected	Located in plaque stagnation areas. (gingival 1/3, pits/fissures, proximal surfaces)	Located mainly in self-cleansing areas. (incisal edges, cups tips, occlusal 1/3 and center of smooth surfaces, may affect entire crown)
Distribution	May affect a single tooth (gingival 1/3, pits/fissures, proximal surfaces)	Multiple teeth involvement (i.e., bilateral or quadrilateral on corresponding (sister) teeth in the same location and with the same shape)

Test and control sites were then selected on unrestored surfaces of some teeth. These included two teeth each with at least one active initial caries (ICDAS 1 or 2), two teeth each with at least one inactive initial caries (ICDAS 1 or 2), and two teeth each with at least one clinically sound surface. If present in these selected teeth, additional active and inactive initial caries, and cavitated lesions were optionally included. The selected lesions/surfaces and their respective tooth numbers were recorded on a separate case report form (CRF).

#### Index test: LC Rinse assessment

The LC Rinse was applied in accordance with the device’s ‘Instructions for Use (IFU)’. Subjects were instructed to swish the rinse vigorously between their teeth for 30 seconds. After removal using a saliva ejector, subjects rinsed with 20 mL of tap water for 10 seconds, which was then removed using a saliva ejector.

Immediately following the water rinse, the examiner performed the LC Rinse assessment. With the examiner wearing a magnifying prism loupe (Designs for Vision, Inc., Bohemia, NY, USA) with Orascoptic™ Ease-In-Shields™ loupes inserts orange protective eye shield, each tooth surface was dried immediately prior to examination with compressed air from the dental air-water syringe. Using an UltraDent Valo™ dental curing light at standard power, held 1-2 inches from the tooth surface under examination, the examiner evaluated the tooth surface while illuminated. Each tooth surface was evaluated for the presence (qualitative positive test response) or absence (qualitative negative test response) of fluorescent illumination (Fig. 1a, b). A dental mirror was used to improve visibility or direct the light source onto the tooth surface. Additional air-drying, water irrigation, suction, or flossing were used to exclude illumination due to residual or pooling of the test rinse.

The selected study teeth were examined first, followed by an examination of the entire dentition for areas of illumination. Results were recorded for the selected teeth and full mouth exams on the appropriate CRF. The investigator repeated the assessment at 10 min, 20 min, and 30 min from the time of rinsing to determine the limits of the time before the test turned negative i.e., the assessment window.

#### Safety monitoring

The clinical examiner conducted a thorough evaluation of the oral soft tissues (OST) before and immediately after the LC Rinse examination. Telephone calls for any adverse events were made 2 and 4 weeks after the clinical encounter.

### Sample size and power calculations

The G*power software version 3.1.9.7 was used to determine the minimum sample size needed to conduct the Chi-Square test of independence. Since two levels of LC Rinse (positive and negative) and two levels of ICDAS (active and inactive) were used for most of the analysis, a 2 × 2 goodness-of-fit contingency table with 1 degree of freedom was used for the analysis. One degree of freedom was obtained by using the (r-1) (c–1) formula for computing degrees of freedom for contingency tables where r = number of columns (or levels of one categorical variable) and c = number of rows (or levels of the second variable). A prior significance level of 0.05, an effect size of 0.3, and a statistical power of 0.9 were used to produce a minimum required sample size of 117 qualified tooth surfaces. Considering that each recruited subject must have at least 6 qualified surfaces (2 sound, 2 with active, and 2 with inactive initial caries), 117 surfaces required approximately 20 subjects. However, 25 subjects were recruited to make room for subjects that may withdraw in the middle of the study.

### Data analysis

All recruited participants completed the study per protocol. Descriptive statistics were performed for all variables. Two-by-two contingency tables were created of the index test, LC Rinse (positive or negative), by active versus combined inactive lesions/sound tooth surfaces as assessed by ICDAS-II examination (reference method), as well as active versus inactive alone and active versus sound alone, and additionally for active versus hypomineralization. The proportion of LC Rinse positive was compared between these groups and a Chi-square test *X*^2^ (1, *N* = 25; α = 0.05) was performed. A diagnostic Odds Ratio (OR) and associated confidence intervals were computed for each comparison. Analyses were performed for both the selected teeth and full dentition assessment. Sensitivity (Se), specificity (Sp), positive and negative predictive values (PPV and NVP), and diagnostic accuracy were also calculated from the contingency table to determine the validity of the new diagnostic tool. The Receiver Operating Characteristic (ROC) Area Under the Curve (AUC) was calculated to compare the diagnostic performance of the different methods.

To determine the duration of LC Rinse assessment positivity in initial caries lesion (“assessment window”) to enable the establishment of a cut-off point for assessment after rinse, a new contingency table was created to compare the assessment at 10 min, 20 min and 30 min to the initial LC Rinse examination. From this contingency table the sensitivity, specificity, and ROC-AUCs were calculated and compared for the different times (0 min to 10 min, 0 min to 20 min, and 0 min to 30 min).

## Results

**Fig. 2:**
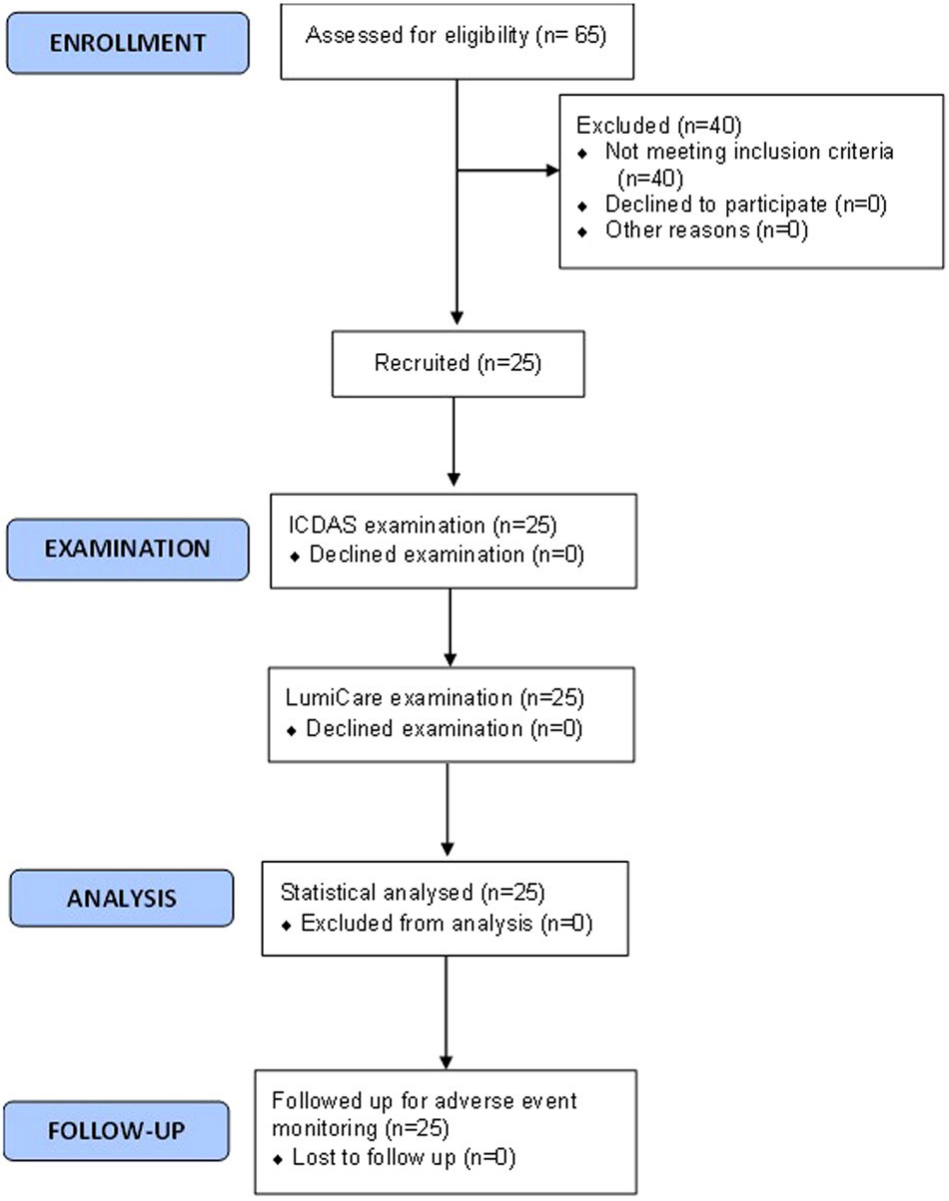
A flow diagram representing the study design.

A flow diagram representing the study design is shown in Fig. 2. The mean age of the participants was approximately 25 ± 13.9 years, 52% were females and more than half of the participants were of the Hispanic race (Table 3). LC Rinse was well-tolerated by the subjects and there were no AEs or any side effects reported during the study and up to 4 weeks after the study.

**Table 3:** Frequency of Analysis on Demographic information and Frequency counts and percentages of different variables from Selected study teeth and Full dentition ICDAS assessment by tooth Surfaces.

**Mean, Standard Deviation and Frequency of Analysis one Variables**		
Characteristic		*M* (SD)
Age		25 (14) *CI* [20, 31]
Gender		
	Male	12 (48%)
	Female	13 (52%)
**Frequency counts and percentages of different variables from Full dentition and Selected teeth ICDAS assessment by Tooth Surfaces**		
Characteristic	ICDAS FULL Dentition	ICDAS SELECTED TEETH
Sound	828 (55%)	71 (32%)
Non-Cavitated-Active	364 (24%)	75 (33%)
Non-Cavitated-Inactive	245 (16%)	53 (24%)
Cavitated	46 (3%)	26 (12%)
Hypomineralization	11 (0.7)	0 (0)

*Mean* Mean, *SD* Standard Deviation, *CI* Confident Intervals.

### Selected study teeth examination: Initial LC Rinse (Index test) versus ICDAS (Reference test) assessments

**Fig. 3:**
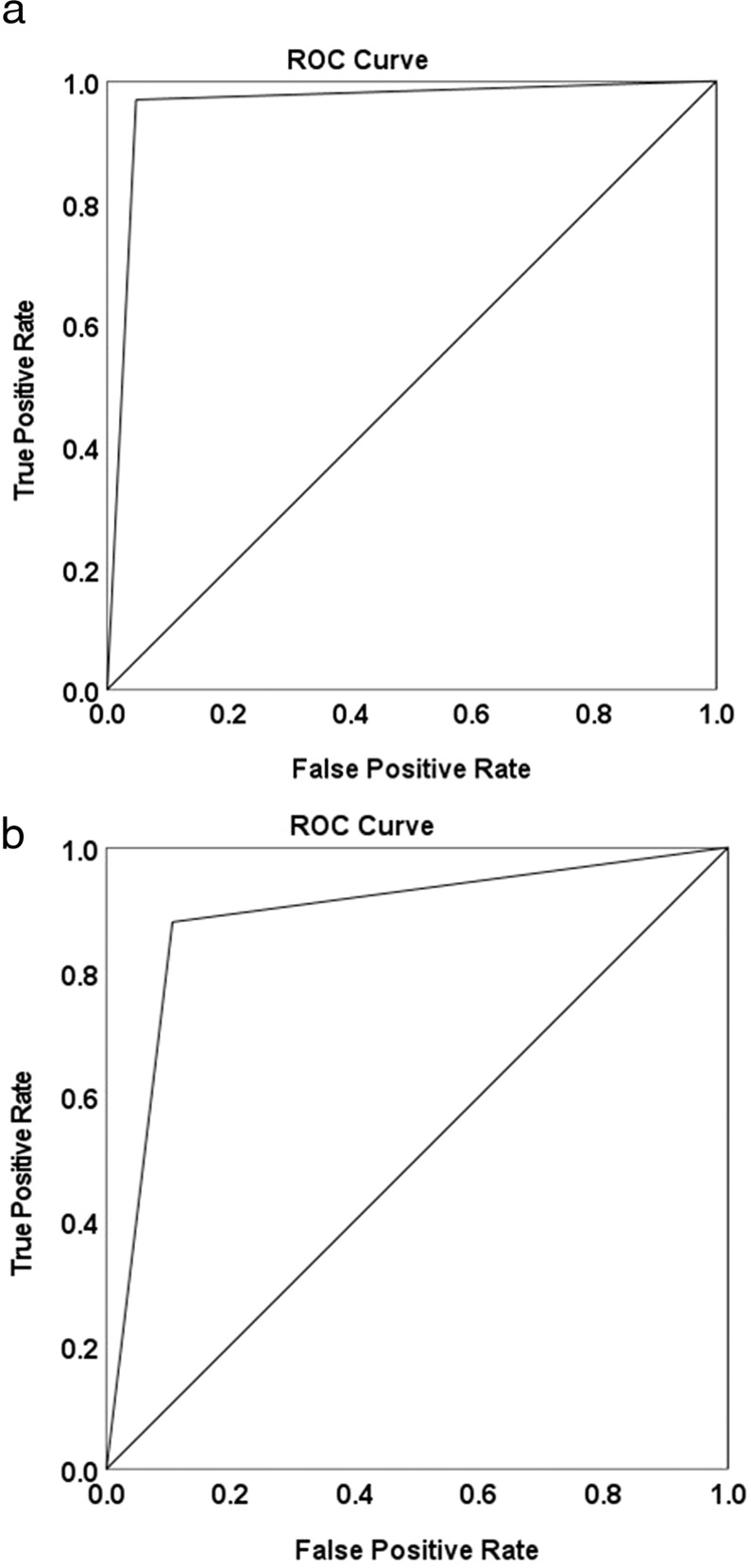
Receiver Operating Characteristic (ROC) for selected teeth and Full dentition assessments. **a** ROC for Selected study teeth assessment (LC Rinse vs ICDAS). Area under the ROC curve: 96.11%. **b** ROC for Full dentition assessment (LC Rinse vs ICDAS). Area under the ROC curve: 88.54%.

Computation of the diagnostic OR, indicated that active caries lesions as assessed by ICDAS examination are 638.6 times (*OR* = 638.6) more likely to have positive LC Rinse test response than sound enamel surfaces or inactive caries lesions, 95% CI [155.65, 2620.19] (Table 4). The Chi-square test indicated a statistically significant association (*p* < 0.01) between these two assessments. Table 4 also shows the proportion of active caries lesions that tested positive for the LC Rinse test (*Sensitivity)*; the proportion of inactive/sound teeth that tested negative for the LC Rinse test (*Specificity)*; the proportion of positive LC Rinse test that are active (*Positive Predictive Value*); the proportion of negative LC Rinse test that is inactive (*Negative Predictive value*); the diagnostic accuracy of the LC Rinse; and the ROC-AUC (Fig. 3a).

**Table 4:** Comparing ICDAS assessment and LumiCare™ Caries Detection Rinse (LC Rinse) assessment.

			ICDAS (reference)		Odds Ratio	Sensitivity	Specificity	Positive Predictive Value	Negative Predictive Value	Diagnostic Accuracy	AUC
**SELECTED STUDY TEETH examination: Initial LumiCare Rinse (Index test) versus ICDAS (reference) assessments**											
Active lesions versus combined Sound/Inactive			Active	Inactive/Sound							
	LC Rinse	+	95	3	638.6 (*X*^*2*^ *p* < 0.01)	0.941	0.976	0.953	0.960	0.960	96.11%
		−	6	121							
		*N*	101	124							
Active lesions versus Inactive only			Active	Inactive							
	LC Rinse	+	69	3	191.7	0.920	0.943	0.958	0.893	0.93	
		−	6	50							
		*N*	75	53							
Active lesions versus Sound surfaces only			Active	Sound							
	LC Rinse	+	69	0	Undefined (Too large due to zero)	0.92	1	1	0.922	0.959	
		−	6	71							
		*N*	75	71							
**FULL DENTITION examination: Initial LumiCare Rinse (Index test) versus ICDAS (reference) assessments**											
Active lesions versus combined Sound/Inactive			Active	Inactive/Sound							
	LC Rinse	+	285	40	60.05 (*X*^*2*^ *p* < 0.01)	0.697	0.963	0.877	0.894	0.89	88.54%
		−	124	1045							
		*N*	409	1085							
Active lesions versus Inactive only			Active	Inactive							
	LC Rinse	+	246	25	18.35	0.675	0.898	0.908	0.651	0.765	
		−	118	220							
		*N*	364	245							
Active lesions versus Sound surfaces only			Active	Sound							
	LC Rinse	+	246	13	130.7	0.676	0.984	0.95	0.874	0.89	
		−	118	815							
		*N*	364	828							
**FULL DENTITION examination: Initial LumiCare Rinse (Index test) versus Clinical Diagnosis (reference)**											
Active lesions versus Hypomineralization			Active	Hypomineralization							
	LC Rinse	+	246	1	20.85	0.675	0.91	0.996	0.078	0.683	
		−	118	10							
		*N*	364	11							

*AUC* Area under the Receiver Operating Curve, *ICDAS* International Caries Detection and Assessment System.

When comparing the response of active initial caries lesions to LC Rinse with that of only inactive lesions, diagnostic OR indicated that active lesions, as assessed by the ICDAS exam, are 191.67 times (*OR* = 191.67) more likely to have positive LC Rinse Test response than inactive lesions, 95% CI [45.73, 803.25]. Additional diagnostic measures are also shown in Table 4.

### Full Mouth: Initial LC Rinse assessment (Index test) versus ICDAS (Reference Test)

Computation of the diagnostic OR comparing the response to LC Rinse of active initial caries lesions with those of sound surfaces or inactive lesions in an unselected sample of teeth indicated that active caries lesions as assessed by ICDAS exam are 60.05 times (*OR* = 60.05) more likely to have positive LC Rinse test response than combined sound surfaces and inactive lesions, 95% CI [41.08, 87.78]. Similarly, active initial caries lesions are 18.35 times (*OR* = 18.35) more likely to have a positive LC Rinse test response than inactive lesions, 95% CI [11.49, 29.30]. Table 4 also shows the Se, Sp, PPV, NPV, and Diagnostic Accuracy for all comparisons of Initial LC Rinse assessment versus ICDAS and ROC-AUC for the full dentition examination (Fig. 3b). Of hypomineralization, only 1 out of 11 was noted to have a positive LC test response and active caries lesions were 20.86 times more likely to have positive LC Rinse test response than hypomineralization.

### Selected study teeth examination: determination of the ‘Assessment Window’

Compared to the initial LC test result as the reference, sensitivity, specificity, and diagnostic accuracy were 0.908, 1.00, and 0.96 at 10 min, 0.755, 1.00, and 0.89 at 20 min, and 0.52, 1.00 and 0.79 at 30 min.

## Discussion

Minimal intervention dentistry (MID) aims to preserve dental tissues first and restore them only when indicated, thus, detection of initial caries is a cornerstone of MID in caries management [28]. However, the current visual-tactile method by which dental practitioners detect and diagnose dental caries, particularly initial caries, is subjective and has limited efficacy in distinguishing between active initial lesions, arrested (inactive) initial caries, and hypomineralization [6, 10, 20–22]. Furthermore, it is well known that the assessment of a lesion’s true activity status in a single visit remains challenging, and the determination of which initial lesions will progress can normally be validated prospectively by serial monitoring over time [29]. Therefore, there is a need for technology to improve the accuracy of visual-tactile caries detection by eliminating subjectivity and enhancing clinician competency to objectively detect and distinguish active initial caries from inactive lesions and hypomineralization. For this reason, we conducted a study evaluating the diagnostic accuracy of an innovative caries-detecting oral rinse, LC Rinse, to potentially augment the ICDAS visual-tactile caries assessment. LC Rinse contains targeted fluorescent starch nanoparticles. It is known that the enamel surface of an active initial caries is microscopically porous but macroscopically sound [30], so when an initial caries is contacted by LC Rinse through mouth rinsing, the fluorescent starch nanoparticles infiltrate micropores of the caries lesion. When this nanoparticle-infiltrated lesion is illuminated with a dental curing lamp, the nanoparticles fluorescently illuminate the caries lesion (positive LC Rinse response) (Fig. 1a, b). LC Rinse does not need expensive equipment that requires maintenance or calibration but only requires a dental curing light, and therefore is cost-effective, user-friendly, and can easily be introduced in everyday practice.

In the present study, the likelihood of ICDAS-assessed active initial caries lesions to fluorescently illuminate with LC Rinse was investigated as compared to sound surfaces and inactive initial caries lesions. Considering the mode of action of the LC Rinse, it was not surprising that the present study showed that the active caries lesions are 639 (60 in full mouth assessment) times more likely to have positive LC Rinse response than sound surfaces and inactive lesions combined and 192 (18 in full mouth assessment) times than inactive lesions only (Table 4). This result confirms the findings of a previous in vitro study that used Two-Photon Microscopy to confirm selective binding and accumulation of cationic fluorescent nanoparticles (size 101 ± 56 nm) within microscopic pores in porous enamel, which were not observed in control samples [23]. The accumulated functionalized fluorescent starch particles were observed to selectively illuminate artificially demineralized lesions, but not healthy tooth surfaces on extracted human teeth [23]. It also has been shown that the caries lesions have a negative surface charge, and therefore cationic nanoparticles can be used for targeting lesions with electrostatic interactions [23]. In another in vitro study, extracted human teeth with demineralized lesions that were subsequently remineralized, exhibited reduction/elimination of fluorescent illumination, thus supporting the negative LC Rinse response by inactive caries lesions in the present study [24].

The greater Se, Sp, PPV, NPV and diagnostic accuracy in selected teeth assessment compared to full dentition assessment is unsurprising. The caries lesions and the sound surfaces in the selected teeth were consciously chosen, thus ensuring the authenticity and certainty of their conditions (active or inactive or sound), while the authenticity and certainty of the full mouth evaluation is subject to more variation and uncertainty. Most conditions are either present or absent over a continuum ranging from small changes to advanced disease, and test sensitivity is often higher in studies in which subjects (in this case, teeth) are selected, as they have more advanced stages of the disease, are often easier to identify, and the diagnosis is certain [31]. Studies where only selected teeth are included, therefore risk selection bias. This study shows that even when all consecutive eligible teeth were included (full dentition), LC Rinse demonstrated high diagnostic accuracy. Furthermore, the full dentition examination, which was considered an exploratory pilot evaluation, was procedurally suboptimal due to practical time limitations because of the repeat evaluations necessary for the second study aim (determination of “assessment window”). Diagnostic accuracy is expected to be even greater with additional care given to the respective examinations.

The result of the present study indicated that the application of LC Rinse vastly improved the sensitivity, specificity, and diagnostic accuracy of visual-tactile caries relative to previous reports (Table 4) [32–34]. This can be attributed to the fact that the fluorescent particles infiltrated and illuminated only the porous surfaces of the active lesions, and not sound surfaces, inactive lesions, or developmental hypomineralization, thereby increasing its specificity. Illumination of active lesions also made them more easily detectable, particularly ICDAS score 1 lesions that are not visible in the presence of saliva [2, 6, 20–22].

When a white spot lesion (WSL) is detected in clinical examination, the differential diagnoses are initial caries lesion and developmental hypomineralization. When a definitive diagnosis of initial caries is made, the next step is confirming the activity stage (active or inactive) of the lesion. For this reason, in the present study, the clinical examiner took these steps to diagnose each WSL as either initial caries or hypomineralization and to decide whether any diagnosed initial caries is still active or arrested (inactive). In this study, active lesions were over 20 times more likely to have a positive LC Rinse response than developmental hypomineralization (Table 4). Thus, LC Rinse differs from other caries detection technologies, in that it facilitates objective detection of caries by its additional ability to distinguish between active caries, inactive caries and hypomineralization.

The idea of using dyes to aid visual-tactile caries detection has persisted for more than 4 decades, but was hindered by the difficulty of clearing the dyes from the micropores in carious tooth tissue after detection [35–37]. This problem is resolved with the LC Rinse as its component nanoparticles are made from a cationic fluorescein-labeled food-grade starch to fluoresce when illuminated by a curing light and then degrade in the oral cavity [23, 24]. Its degradation is confirmed in the present study by the gradual decrease in Sensitivity and diagnostic accuracy over time, indicative of the particles and their illumination degrading and washing away. Considering that the clinical examiner was able to complete the full mouth examination within 10 min, it can be advised confidently that the LC Rinse will remain effective at 10 min following rinsing. As such, the lapse time between the start and end of the assessment would not significantly affect the effectiveness of the LC Rinse.

The remineralization of initial caries, an integral part of caries management, is an essential treatment strategy in MID. However, to date there is no available clinical tool to objectively monitor the efficacy of therapeutic materials used in the arrestment/remineralization of initial caries lesions, particularly on coronal tooth surfaces. The present study indicated a statistically significant greater likelihood of illumination in active initial caries compared to inactive lesions, with the active lesions being 192 times more likely to have positive LC Rinse Test response than inactive caries in selected teeth and 18 times increased likelihood in full mouth examination. The ability to efficiently distinguish between active and inactive (arrested) caries lesions makes LC Rinse a useful clinical tool to enable effective monitoring of remineralization treatment in caries management, as demonstrated in vitro with extracted human teeth with demineralized lesions that were subsequently remineralized and exhibited reduction in fluorescent illumination by LC Rinse [24]. In theory, change in lesion activity may be a surrogate indicator of caries progression or remineralization treatment efficacy, which could replace longer-term outcome metrics such as a change in ICDAS score, or need for surgical treatments, particularly when combined with AI/computer vision [25]. This potential to monitor progression or remineralization over time should be investigated and confirmed in further studies.

There were no adverse effects reported by the subjects or observed during clinical examination, which is attributed to the use of food-grade starch as the base polymer, making the particles nontoxic and biodegradable upon exposure to salivary amylase [23, 24].

Although proximal surfaces were not included in the examination, which may be regarded as a study limitation, LC Rinse was observed to illuminate proximal initial caries lesions that extended to have portions of the lesion that were visible on buccal or lingual surfaces. This study demonstrated a successful Phase II and pilot Phase III evaluation of the diagnostic accuracy of LC Rinse, and we suggest future studies with suitable sample sizes per appropriate calculations and possibly multicenter studies as future next steps to validate the technology. It was observed that the positioning and angulation of the curing light might impact the accuracy of the caries detection by influencing the fluorescence of the lesions. Thus, some practice and/or training may be required to minimize the possibility of false negatives or false positives, which would affect the Se, Sp, PPV, NPV, and diagnostic accuracy, and may lead to undertreatment or overtreatment.

## Conclusion

The present clinical trial demonstrated:

- clinical accuracy of the LC Rinse in enhancing the detection of active initial caries in a clinical setting, with high sensitivity, specificity, and diagnostic accuracy, and as such, can be a valuable clinical tool for dental practitioners as an aid to caries detection and diagnosis.
- that when the examination is completed within 10 min of rinsing with LC Rinse, active non-cavitated caries lesions are more likely to show LC Rinse fluorescent illumination than inactive non-cavitated caries lesions or healthy (sound) tooth surfaces, and as such can serve as a valuable device for distinguishing between active and inactive caries lesions.
- as time elapses, the sensitivity, specificity, and diagnostic accuracy of the LC Rinse in enhancing the detection of caries disease diminishes, and as such it is advised that the assessment be completed within 10 min of application of the rinse.
